# Moderator role of vitamin D concentrations on the association between metabolic syndrome and C-reactive protein among adults

**DOI:** 10.20945/2359-3997000000272

**Published:** 2020-06-19

**Authors:** Angelica Scherlowski Fassula, David Gonzalez-Chica, Marui Corseuil Giehl, Diego Augusto Santos Silva, Francieli Cembranel, Yara Maria Franco Moreno

**Affiliations:** 1 Programa de Pós-Graduação em Nutrição Universidade Federal de Santa Catarina Florianópolis SC Brasil Programa de Pós-Graduação em Nutrição, Universidade Federal de Santa Catarina, Florianópolis, SC, Brasil; 2 Faculdade de Medicina de Adelaide Universidade de Adelaide Adelaide Austrália Disciplina de Clínica Geral, Faculdade de Medicina de Adelaide, Universidade de Adelaide, Adelaide, Austrália; 3 Escola Clínica Rural de Adelaide Universidade de Adelaide Adelaide Austrália Escola Clínica Rural de Adelaide, Universidade de Adelaide, Adelaide, Austrália; 4 Departamento de Ciências da Saúde Universidade Federal de Santa Catarina Araranguá SC Brasil Departamento de Ciências da Saúde, Universidade Federal de Santa Catarina, Araranguá, SC, Brasil; 5 Departamento de Educação Física Universidade Federal de Santa Catarina Florianópolis SC Brasil Departamento de Educação Física, Universidade Federal de Santa Catarina, Florianópolis, SC, Brasil; 6 Departamento de Nutrição Universidade Federal de Santa Catarina Florianópolis SC Brasil Departamento de Nutrição, Universidade Federal de Santa Catarina, Florianópolis, SC, Brasil

**Keywords:** Metabolic syndrome, vitamin D, C-reactive protein, insulin resistance, epidemiological research design

## Abstract

**Objective:**

To evaluate the association between MetS, its components and insulin resistance (IR) with 25(OH)D and hsCRP. The moderator role of 25(OH)D in the association of MetS, its diagnostic components and IR with hsCRP were also explored.

**Materials and methods:**

A cross-sectional study (2014/2015) with a population-based cohort in Southern Brazil (n = 605). Metabolic syndrome (MetS) diagnosis was defined based on the Joint Interim Statement, while the Homeostasis Model Assessment of insulin resistance (IR) (HOMA-IR) was used for determining IR. Serum concentrations of 25-hydroxy vitamin D [25(OH)D] (ng/mL) and high sensitivity C-reactive protein (hsCRP) (mg/L) were evaluated following standard protocols. 25(OH)D was categorized as sufficiency (>30 ng/mL), insufficiency (20-30 ng/mL) or deficiency (<20 ng/mL) to test its moderator role. Multiple linear regression was used to test the associations. The results were adjusted for possible confounders.

**Results and discussion:**

Hypertriglyceridemia and IR were associated with lower 25(OH)D concentrations. However, except for systolic blood pressure, other MetS components and IR were associated with higher hsCRP. The association between elevated waist circumference (WC) and hsCRP was moderated by the 25(OH)D concentrations. The hsCRP median concentrations were more than two times higher among those with elevated WC and 25(OH)D insufficiency or deficiency. In this study, inadequate concentrations of 25(OH)D increased the adverse relationship between elevated WC and inflammation. 25(OH)D concentrations could be incorporated into the clinical protocols for monitoring individuals with abdominal obesity to identify those at a higher risk of complications.

## INTRODUCTION

Metabolic syndrome (MetS) is a condition that combines several cardiometabolic risk factors, including abdominal obesity, hypertension, dyslipidemia, hyperglycemia and insulin resistance (IR). MetS is associated with the development of diabetes mellitus, cardiovascular disease (CVD) and their complications, including hospitalizations and deaths ( 1 ).

MetS is characterized by the release of free fatty acids, which promote chain reactions in different tissues, modifying glycemic markers, lipoproteins, blood pressure concentrations and IR ( 1 ). These metabolic abnormalities are associated with chronic inflammation of low intensity, including increased synthesis of C-reactive protein (CRP), which is a marker of cardiovascular risk ( 2 ).

In parallel, hypovitaminosis D has also been associated with systemic inflammation and MetS ( 3 - 5 ). The Framingham Offspring Study conducted a study between 1997-2001 to corroborate data provided by in vitro studies suggesting a protective effect of vitamin D (25(OH)D) on inflammatory markers, but found a null association between concentrations of this vitamin and high-sensitivity CRP (hsCRP) concentrations ( 3 ). Nonetheless, an Australian study including healthy individuals found an inverse association between 25(OH)D and hsCRP ( 4 ), while a German population-based study involving individuals aged 25-88 verified a U-shaped relationship between these variables ( 5 ). In the German study, hsCRP was lower among those with a 25(OH)D concentration between 21-25 ng/mL, while higher values of hsCRP were observed when the concentrations of 25(OH)D were below 21 or above 25 ng/mL ( 5 ).

Moreover, an Iranian randomized controlled trial (RCT) found that vitamin D supplementation in diabetic adults with coronary artery disease and deficiency of vitamin D reduced glycemia and CRP (n = 60) ( 6 ). However, that association was not consistent in studies including overweight or obese adults ( 7 ).

The possible moderator role of 25(OH)D in the association between MetS and IR with CRP has been barely explored in population-based samples from middle or low-income countries. This study is relevant considering the abrupt increase in the prevalence of obesity, diabetes, and other non-communicable diseases that these countries are facing ( 8 ).

Therefore, this study was designed to investigate the association of MetS, its diagnostic components and IR with 25(OH)D and hsCRP concentrations by using a population-based cohort of adults in Southern Brazil. The moderator role of 25(OH)D in the association between MetS and hsCRP was also explored.

## MATERIALS AND METHODS

### Study design

This study constituted a cross-sectional analysis of the third wave of the EpiFloripa Adults Cohort Study (2014/15), a populational-based study started in 2009 with a representative sample of adults, living in Florianópolis, capital of Santa Catarina State, Southern Brazil.

The random sampling process was used in the baseline recruited 1,720 individuals and was performed in two phases. Firstly, 63/420 census tracts (census units) were systematically selected in each decile of family income, and subsequently, 1,134/16,755 households were systematically selected in these sectors. All individuals between 20 and 59 years old were considered eligible, except those who had been amputated, bedridden, hospitalized, or affected by severe mental or physical health illnesses that could compromise anthropometric measurements, or the quality of the information obtained during the interviews.

The third wave of EpiFloripa Adults study was approved by the Ethics Committee on Human Research from State University of Santa Catarina (protocol nº 724.824; CAEE number 31020214.4.0000.0118). All participants signed an informed consent form.

### Participants

**Figure 1:**
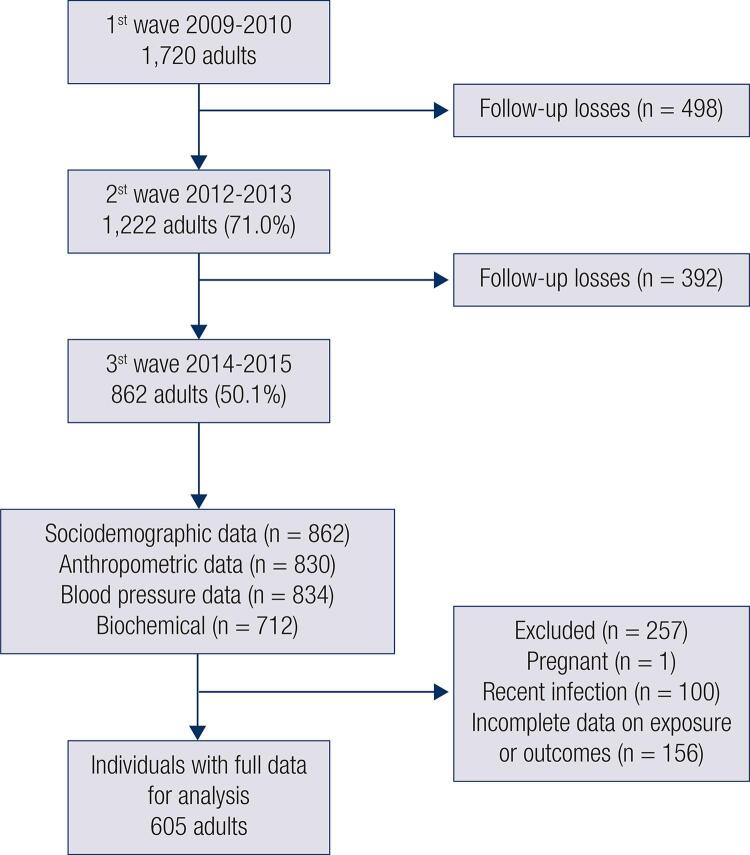
Flowchart of EpiFloripa Adults cohort study (2009-2014).

The baseline of the EpiFloripa Adults Study (2009) intended to estimate the prevalence of several health outcomes by using face-to-face interviews at the household of the participants. The present study was conducted between August 2014 and June 2015, with all adults included in the baseline traced (third wave of the cohort, Figure 1 ) and having their anthropometric measurements and blood pressure assessed at the Laboratory of Anthropometry of the Federal University of Santa Catarina, where blood samples were also collected for laboratory tests.

Of the 1,720 individuals included in the baseline, 50.1% were located and interviewed in 2014/15. The final sample included for analysis (complete data for exposure and outcomes, excluding pregnant women (n = 1) and those with a recent infectious disease (n = 100) was 605 individuals. The power of the study was estimated a posteriori, considering an exposure prevalence of 35% (MetS) and the parameters of the outcomes [25(OH)D 24.7 ± 8.1 and hsCRP_ln_ 0.25 ± 1.15]. Based on the available sample size, this study was able to detect mean differences of at least 2.8 ng/mL and 0.40 mg/L, respectively (alpha = 5%, power = 80%, design effect = 1.5).

### Data collection

A questionnaire was used in each cohort wave to collect data on sociodemographic characteristics, lifestyle (i.e. current smoking, physical activity level), self-reported conditions, medication use and other health variables. Physical activity was evaluated using a validated questionnaire from the Surveillance of Risk Factors and Protection for Chronic Diseases by Telephone Enquiry (Vigitel) ( 9 ).

Standardized techniques were used to measure weight (kg), height (m) and waist circumference (WC) (cm). Anthropometric measures were assessed twice and the mean of two measurements was used in the study. Weight was measured using portable digital scales (Marte^®^ LC 200 pp, São Paulo, Brazil), with 150 kg capacity and 100 g resolution. Height was measured using a stadiometer (Altura Exata^®^, Santa Efigênia, Brazil) with 1 mm resolution and 250 cm capacity. WC was measured using an inelastic anthropometric tape (Sanny^®^, São Bernardo do Campo, Brazil) with 1 mm resolution and 200 cm capacity. Data collection was performed after equipment calibration and interviewer training (including standardization of anthropometric measurements according to technical errors of measurement (TEM) intra- and inter-observer) ( 10 ).

Blood pressure was also measured twice using a digital wrist sphygmomanometer (Techline^®^, São Paulo, Brazil) and following the protocol of the Brazilian Society of Cardiology ( 11 ). A rest interval of at least 15 minutes before the first measurement and between measurements was considered. When the difference between measurements was greater than 20 mmHg for systolic blood pressure (SBP) or 10 mmHg for diastolic blood pressure (DBP), a third blood pressure measurement was performed to replace the highest value. The mean of the two lowest blood pressure measurements was considered for analysis.

Blood samples were collected by peripheral venipuncture after 8-10 hours fasting and then stored, transported and analyzed following a standard protocol. Fasting serum glucose concentration was measured by adapting the hexokinase-glucose-6-phosphate dehydrogenase method using the cartridge kit Flex^®^ Reagent Cartridge GLUC and the autoanalyser Dimension^®^ Clinical Chemistry System (Siemens Healthcare Diagnostics Inc. Newark, United States). Fasting serum insulin concentration was measured by the particle chemiluminescence method (Centauro^®^ Siemens Healthcare Diagnostics Inc., Newark, United States).

Triglycerides serum concentration was measured by automated endpoint bichromatic colorimetric enzymatic method, using cartridge kits (Flex^®^ Reagent Cartridge CHOL and TGL, Newark, United States). HDL-cholesterol serum concentration was measured by selective accelerator detergent method (Flex^®^ Reagent Cartridge AHDL, Newark, United States).

Finally, 25(OH)D serum concentration was measured by the Particle Chemiluminescence method (Centauro^®^ Siemens Healthcare Diagnostics Inc., Newark, United States), while hsCRP serum concentration was measured using immunonephelometry (BN II^®^, Siemens Healthcare Diagnostics Inc., Newark, United States).

### Definition of exposure and outcome variables

MetS diagnosis was based on the Joint Interim Statement JIS 2009 criterion ( 12 ). Individuals were considered positive for MetS when they presented at least three altered components of: 1) elevated WC (> 90 cm for men or > 80 cm for women); 2) high blood pressure (SBP ≥ 130 mm/Hg or DBP ≥ 85 mm/Hg); 3) hyperglycemia (fasting blood glucose ≥ 100 mg/dL); 4) elevated triglycerides (≥ 150 mg/dL), and; 5) reduced HDL-cholesterol (< 40 mg/dL in men or < 50 mg/dL in women). Regardless of the laboratory results, individuals who reported the use of some medication for controlling their blood pressure, glycemia, or lipid concentrations were considered as positive for high blood pressure, hyperglycemia or dyslipidemia, respectively.

To determining IR, the mathematical model Homeostasis Model Assessment of Insulin Resistance (HOMA-IR) was used (HOMA-IR = fasting glycemia (mmol/L) x fasting insulin (uU/mL)/22.5). The adopted cut-off point for the Brazilian population was a value above 2.7 ( 13 ).

Due to its non-normal distribution, hsCRP (in mg/L) was transformed into its natural logarithm (hsCRP_ln_) and analyzed as a continuous variable. 25(OH)D concentrations were analyzed as a continuous variable and additionally classified according to the Endocrine Society clinical practice guideline and Recommendations of the Brazilian Society of Endocrinology and Metabology ( 14 , 15 ) as sufficiency (> 30 ng/mL), insufficiency (20-30 ng/mL) or deficiency (< 20 ng/mL).

### Confounding factors

These variables were selected based on the evidence of previous studies that have associated MetS, 25(OH)D deficiency and increased hsCRP with age, sex, education level, smoking, physical activity, solar radiation levels and body mass index (BMI, based on measured weight and height) ( 16 ). Physical activity level was evaluated by combining intensity, frequency and duration of leisure physical activity. Individuals were considered physically active when they reported moderate-intensity physical activity of 30 or more minutes at least five days/week or vigorous-intensity physical activities for at least 20 minutes in three or more days/week ( 9 ). Solar radiation was defined based on the interview month (blood collection), as higher radiation levels for the Southern hemisphere occur between September and March, and lower levels from April to August.

### Statistical analysis

Asymmetric continuous variables were presented as medians and interquartile range, and symmetric variables as mean and standard deviation. Categorical variables were presented as percentage (%).

The comparison of the sample characteristics between those with or without MetS was performed using the chi-square test for categorical variables and T-test for continuous variables.

Multiple linear regression was used to test the association between MetS, its diagnostic components and IR with the 25(OH)D and hsCRP_ln_ concentrations. These results were expressed as regression coefficients (β) with their respective 95% confidence interval (95% CI). Results for hsCRP_ln_ were presented in its exponential form (β_exp_) and interpreted as a risk ratio (times higher/lower hsCRP concentration among exposed compared to the geometric mean observed in non-exposed).

All analyses were adjusted for sex, age, education level, smoking, physical activity level and BMI. The solar radiation level was an additional confounder considered in adjusted analysis when the 25(OH)D was investigated as the outcome. The variance inflation factor (VIF) was investigated as an indicator of over-adjustment and multicollinearity between the explanatory variables.

The possible moderator role of 25(OH)D in the association of MetS, its diagnostic components and IR with hsCRP was investigated by introducing multiplicative terms between 25(OH)D status and hsCRP in the regression models. A p-value < 0.10 for interaction was considered as suggestive of heterogeneity of the effects. In this case, results were presented graphically as the median and interquartile range of hsCRP (estimated based on quantile regression models and adjusted for confounders) among those with or without MetS/IR, stratified according to the 25(OH)D concentrations (sufficiency, insufficiency or deficiency).

All statistical analyses were performed in the STATA^®^ 14.0 statistical software (Stata Corp., College Station, Texas, USA), considering the clusters and using sampling weights (probability of selection in the baseline and probability of being located in 2014/15).

## RESULTS

The sample characteristics at baseline (2009) and their comparison with those included in the study (n = 605) or with MetS are described in Table 1 . The proportion of males at baseline was slightly lower than females and 77.4% had finished their primary education level (> 8 years of education). Around 20% were current smokers, 15% were physically active, and 39% had elevated WC. The distribution of these characteristics among those located in 2014/15 and included for analysis resembled the distribution of the original cohort (less than five percentage point difference for categorical variables and < 10% difference for the mean age). Among individuals with MetS (prevalence 35%, 95% CI 30.0-39.6), there was a higher proportion of males, older individuals, with a lower education level or elevated WC that their peers, but no difference was observed according to physical activity level or smoking status.

**Table 1:** Sample characteristics in the baseline (2009) and comparison with those analyzeda in the study and those with metabolic syndrome (2014/15)

Variables^b^	Baseline (2009)	Third wave (2014/15)^a^	

	(N = 1720)	Analyzed (n = 605)	With MetS (n = 210)

	% Mean ± SD	% Mean ± SD	% Mean ± SD
Gender (males)	44.5	42.5	49.9*
Age (years)	37.7 ± 11.6	39.6 ± 11.4	44.6 ± 10.7*
Education level			
0-8 years	22.7	20.5	28.7*
9-11 years	33.6	30.3	26.8
≥12 years	43.8	49.3	44.5
Current smokers	19.4	15.4	17.5
Physically active^c^	15.4	15.8	13.0
Elevated waist circumference^d^	39.4	43.8	75.5*

* P-value < 0.05 for the comparison between those analysed with or without metabolic syndrome.

^a^ Including individuals located in 2014/15 with complete data for the exposure and outcome variables, but excluding pregnant women (n = 1) or those with a recent infectious disease (n = 100).

^b^ Variables considering characteristics of the sample in the baseline (2009).

^c^ ≥ 30 minutes of moderate-intensity physical activity in at least five days/week or ≥ 20 minutes of vigorous-intensity physical activity in three or more days/week (leisure physical activity).

^d^ Waist circumference > 90.0 cm in males or > 80 cm in female.

Table 2 shows the prevalence of the MetS components and IR and its association with 25(OH)D and hsCRP_ln_ concentrations. Elevated WC and high blood pressure were the most prevalent components of MetS, while 29.1% (95% CI 25.1-33.5) were positives for IR. The mean 25(OH)D in the sample was 24.7 ± 8.1 ng/mL and was lower among those with MetS or IR. Among the MetS components, hypertriglyceridemia was the only variable associated with lower 25(OH)D concentrations. The median hsCRP was 1.34 mg/L (interquartile range 0.60;3.52), and it was 77% higher among those with MetS and twice higher among those with IR. Except for higher SBP, all other MetS components were associated with higher hsCRP. The mean VIF ranged between 1.3 and 1.5 in all analyses, indicating no multicollinearity between the explanatory variables in the final models.

**Table 2:** MetS prevalence and its components, insulin resistance and adjusted association with 25(OH)D and hsCRP concentration (n = 605)

Variables	%	25(OH)D^a^		hsCRP_ln_^b^	

		β_exp_	(95%CI)	β_exp_	(95%CIexp)
Metabolic syndrome^c^	35.0	-0.75	(-2.30; 0.81)	1.46	(1.18; 1.81)**
Elevated WC	47.6	-0.41	(-1.86; 1.04)	2.01	(1.62; 2.51)***
Elevated SBP	50.8	-0.21	(-1.60; 1.18)	1.18	(0.99; 1.41)
Elevated DBP	46.1	-1.09	(-2.75; 0.58)	1.42	(1.17; 1.73)**
Elevated fasting glucose	17.2	-1.43	(-3.33; 0.46)	1.40	(1.10; 1.79)**
Elevated triglycerides	20.5	-3.12	(-4.88; -1.37)**	1.53	(1.20; 1.97)**
Low HDL	36.2	-0.43	(-1.77; 0.90)	1.54	(1.23; 1.93)***
Insulin resistance^d^	29.1	-2.00	(-3.58; -0.41)*	1.75	(1.41; 2.17)***

β = regression coefficient representing the mean difference in 25(OH)D concentrations between exposed and non-exposed.

β_exp_ = regression coefficient in exponential form, interpreted as a risk ratio (times higher/lower hsCRP concentration among exposed compared to the geometric mean among non-exposed).

P-value: * < 0.05; ** < 0.01; *** < 0.001.

CI: confidence interval; 25(OH)D: 25-hydroxyvitamin D; hsCRP_ln_: natural logarithm of the high-sensitivity C-reactive protein; WC: waist circumference; SBP: systolic blood pressure; DBP: diastolic blood pressure; HDL: high-density lipoproteins.

^a^ Mean 25(OH)D 24.7 ± 8.1 ng/mL. Results adjusted for gender, age, education level, physical activity level, smoking, solar radiation level and body mass index.

^b^ Median hsCRP 1.34 mg/L (interquartile range 0.60;3.52). Results adjusted for gender, age, education level, physical activity level, smoking and body mass index.

^c^ MetS: according to the Joint Interim Statement JIS 2009 criterion ( 18 ).

^d^ HOMA-IR: calculated according to Matheus et al. ( 19 ), and cut off Geloneze et al. (>2.7) ( 21 ).

**Figure 2:**
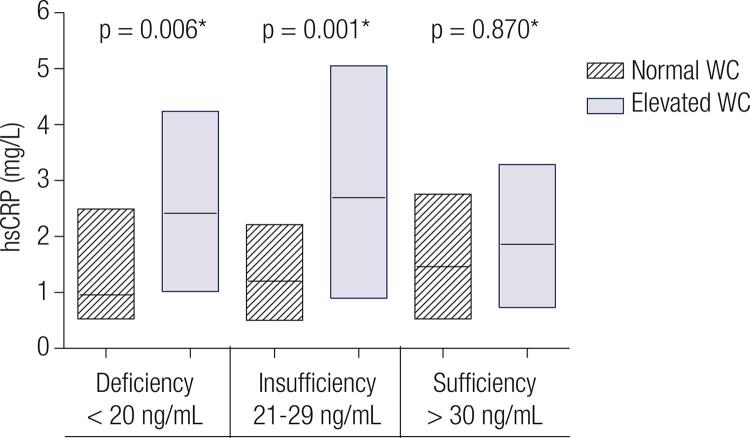
Adjusted association between WC and hsCRP, stratified by 25(OH)D status. Results are adjusted for sex, age, education level, physical activity level, smoking, solar radiation level and body mass index. Central lines represent the adjusted medians. Lower and upper lines represent the adjusted interquartile ranges [p25-p75]. Elevated WC: waist circumference > 90 cm in males and > 80 cm in females.

When the possible moderator role of 25(OH)D was investigated, there was evidence of effect modification only for the association between elevated WC and hsCRP (p-value for interaction 0.03; Figure 2 ). The individuals with normal WC showed similar hsCRP median concentrations in all 25(OH)D status (0.96 mg/L for deficiency, 0.91 mg/L for insufficiency and 1.10 mg/L for sufficiency). However, the individuals with elevated WC and 25(OH)D status of deficiency and insufficiency showed hsCRP median concentrations 2.00 mg/L and 2.14 mg/L respectively, while in sufficiency the hsCRP median was 1.57 mg/L.

## DISCUSSION

This population-based study conducted in Southern Brazil verified that only hypertriglyceridemia and IR were associated with lower 25(OH)D concentrations. On the other hand, except for higher SBP, all other MetS components and IR were associated with higher hsCRP concentrations. Nevertheless, hypovitaminosis D was an effect modifier of the association between elevated WC and hsCRP.

The relationship between MetS, IR and hsCRP is consistent with the available literature ( 17 ). A population-based Iranian study with 3,285 adults showed that higher hsCRP concentrations were associated with all components of MetS, except HDL (β 0.049; 95% CI -0.050; 0.148) ( 2 ). In a Dutch study including 1,165 adults with abdominal obesity, a direct trend association was observed between the number of MetS components and hsCRP ( 17 ). The same study verified that WC and triglycerides were independently associated with hsCRP (β = 0.03 95% IC 0.02;0.03 and β = 0.21 95%IC 0.10;0.32, respectively) ( 17 ). In a Japanese prospective study (n = 1,074), the association between CRP and IR was less evident among adults with normal BMI (β 0.93 95% CI 0.86; 1,00) than among those with overweight (β 1.28 95% CI 1.17; 1,41) or obesity (β 2.13 95% CI 1.98; 2.29) ( *p-value* for interaction < 0.001) ( 18 ).

However, the relationship between MetS, IR and 25(OH)D is not consolidated. In a large population-based cohort with black and white North American adults (n = 28,026), 25(OH)D concentration was inversely associated with WC (β = -2.3; P < 0.001) and IR (β = -0.13; p < 0.001) after adjustment for socioeconomic status, history of diabetes, stroke, lifestyle habits, the season of the blood draw and laboratory parameters ( 19 ). Another population-based study in the United States (n = 7,674) also verified that 25(OH)D sufficiency was associated with 18% risk reduction of MetS (prevalence ratio 0.82 95% CI 0.74-0.91) and 30% risk reduction of IR (prevalence ratio = 0.70 95% CI 0.59-0.84) ( 20 ). Nonetheless, a population-based Iranian study (n = 846) with adults verified a null association between MetS and 25(OH)D (β = -2.85; p = 0.159), even when the MetS components were assessed separately. However, the authors argue that the lack of association could be due to the high prevalence of hypovitaminosis D in the sample, which could interfere with the test power ( 21 ). In this study, hypertriglyceridemia was associated with lower 25(OH)D concentrations. Previous studies have suggested that this relationship might be explained by increased CRP concentrations and abnormal adipocyte function, increasing serum concentrations of free fatty acids and abdominal obesity ( 22 ). However, our results were adjusted for BMI, indicating other factors different from obesity that would explain the relationship between hypertriglyceridemia and 25(OH)D concentrations.

Regarding the moderator role of 25(OH)D in the association between elevated WC and hsCRP, some hypotheses could explain this finding ( 16 , 23 - 28 ). First, the mechanisms associated with lipogenesis were observed in primary-cultured human adipocytes, in which 1α25(OH)_2_D_3_ exerts an inhibitory effect on adipocytes basal lipolysis ( 23 ). In male Wistar rats, it was also demonstrated that 1α-hydroxylase may have a role in the regulation of adipocytes growth and metabolism ( 24 ).

In a North American RCT with black adults (n = 292), baseline hsCRP adjusted for obesity (BMI ≥ 30 kg/m^2^) was inversely associated with baseline 25(OH)D concentration, which allows inferring associations between obesity, inflammation and vitamin D status ( 25 ). The association of 25(OH)D with WC (β = -0.11 95% CI -0.16; -0.06) and hsCRP with WC (β 2.40 95% CI 1.71; 3.08) was explored in a North American study conducted in a community-based sample of adults and older adults (n = 4,010). The authors verified that the effect of 25(OH)D on WC was mediated by CRP, adiponectin and leptin (mediation attributable these mediators = 23%) ( 19 ).

According to a meta-analysis with 13 studies, supplementation with vitamin D in obese and overweight subjects does not have significant changes on hsCRP concentrations (standardized mean difference -0.11 95% CI -0.27; 0.17) and other inflammatory biomarkers, such as tumor necrosis factor-alpha (TNF-α) and interleukin 6 (IL-6) ( 26 ).

The modulation role of 25(OH)D in hsCRP was discussed in a meta-analysis including 13 studies, in which it was demonstrated that supplementation with vitamin D is beneficial for reducing hsCRP (standardized mean difference -0.45 95% CI -0.77; -0.14) in adults and older adult subjects with a diagnosis of diabetes mellitus ( 27 ). Also, a meta-analysis with 81 clinical trials showed a significant reduction in hsCRP (standardized mean difference -0.20 95% CI -0.34; -0.06) after a minimum of 3 months of vitamin D in adults and older adults with different nutrition status BMI ( 28 ).

However, it is recommended a careful interpretation of 25(OH)D status in inflammatory states. Although conducting measurements of serum total 25(OH)D concentrations is the best way to assess vitamin D status in the general population, it might not accurately indicate the vitamin D status during illness ( 29 ). Therefore, the inverse correlation between 25(OH)D and CRP can be more pronounced in patients with inflammatory diseases in comparison to patients with non-inflammatory diseases ( 30 ). The absence of linearity in the associations of CRP and 25(OH)D were described in a recent cross-sectional study from National Health and Nutrition Examination Survey (NHANES). The authors observed that in metabolic diseases, specifically in diabetes, the nonlinear associations of CRP with 25(OH)D had weakening tendency as 25(OH)D increased ( 31 ).

Our findings are relevant for public health policies among countries facing a rapid increase in the prevalence of overweight and non-communicable chronic diseases. During the waves of the EpiFloripa study (2009-2015) the prevalence of overweight among adults in Brasil increased from 46.0% to 53.9%, while obesity raised from 14.3% to 18.9% ( 32 ). As a consequence, the main public health policy implemented in Brazil during this period was The Strategic Action Plan to Tackle Noncommunicable Diseases (NCDs), which was launched in 2011 and included strategies to monitor these conditions, improve lifestyle, and reduce the prevalence and consequences of NCDs ( 33 ). Some positive signs of change were observed over time, including an increase in physical activity levels, fruit and vegetable consumption, reduction of the consumption of sweet drinks and smoking, while the prevalence of obesity, diabetes, and hypertension seems to be increasing slowly in the last few years ( 32 ). Despite the health policies included in The Strategic Action Plan to Tackle NCDs, promoting healthy environments and obesity reduction are considered a good model, they also need to consider activities focused on monitoring and treating factors that influence the complex pathophysiology linking obesity, inflammation and NCDs risk, meeting the principles of synergy and interaction ( 34 ).

The major strengths of the study are the use of a population-based sample from a Latin American country and the validated protocols used for data collection. Some limitations have to be recognized. First, causal inferences are affected by the cross-sectional analysis. Second, the use of vitamin D and medications were not evaluated in the third wave. However, medications for blood glucose control, blood pressure and dyslipidemia that characterizes continuous use for blood glucose, blood pressure and dyslipidemia control were collected in the first wave, and the use of these data minimizes possible biases of MetS under diagnosis by JIS 2009 criteria in the third wave. The use of first wave data minimizes possible biases of MetS underdiagnosis by JIS 2009 criteria. Third, considering that more than 90% of circulating 25(OH)D in human serum is protein bound, changes in the vitamin D-binding protein and albumin concentrations can alter measured total 25(OH)D concentrations without influencing its free concentrations ( 35 ). Although the albumin serum concentrations were not measured in the EpiFloripa study, those individuals with recent infections or clinical conditions that may influence albumin concentrations were excluded from the analyzes to minimize this source of bias.

In summary, this study observed an association between hypertriglyceridemia and IR with 25(OH)D and the association between MetS, its diagnostic components and IR with hsCRP. The results still demonstrated that the association of abdominal obesity (evaluated by WC) and inflammation might be moderated by vitamin D status. Additionally, these findings suggest that 25(OH)D concentrations could be incorporated into the clinical protocols for monitoring individuals with abdominal obesity to identify those at a higher risk of NCDs and their complications.
